# NLRX1: Versatile functions of a mitochondrial NLR protein that controls mitophagy

**DOI:** 10.1016/j.bj.2023.100635

**Published:** 2023-08-11

**Authors:** Paul Y. Bi, Samuel A. Killackey, Linus Schweizer, Stephen E. Girardin

**Affiliations:** a Department of Laboratory Medicine and Pathobiology, University of Toronto, Toronto, Ontario, Canada; b Department of Immunology, University of Toronto, Toronto, Ontario, Canada

**Keywords:** Mitochondria, NLRX1, Mitophagy, Nod-like receptors

## Abstract

NLRX1 is a member of the of the Nod-like receptor (NLR) family, and it represents a unique pattern recognition molecule (PRM) as it localizes to the mitochondrial matrix in resting conditions. Over the past fifteen years, NLRX1 has been proposed to regulate multiple cellular processes, including antiviral immunity, apoptosis, reactive oxygen species (ROS) generation and mitochondrial metabolism. Similarly, in vivo models have shown that NLRX1 was associated with the control of a number of diseases, including multiple sclerosis, colorectal cancer and ischemia-reperfusion injury. This apparent versatility in function hinted that a common and general overarching role for NLRX1 may exist. Recent evidence has suggested that NLRX1 controls mitophagy through the detection of a specific “danger signal”, namely the defective import of proteins into mitochondria, or mitochondrial protein import stress (MPIS). In this review article, we propose that mitophagy regulation may represent the overarching process detected by NLRX1, which could in turn impact on a number of diseases if dysfunctional.

## Highlights

• NLRX1 controls mitophagy through the detection of a specific “danger signal”: mitochondrial protein import stress (MPIS).

**At a glance of commentary**

**Scientific background on the subject**

*Vibrio parahaemolyticus* is the leading cause of gastrointestinal illness following consumption of raw or undercooked seafood. This bacterium is also a significant biohazard for the aquaculture industry, causing early mortality syndrome in shrimp. Rapid detection of *V. parahaemolyticus* and other pathogens is crucial to ensuring food safety and public health.

**What this study adds to the field**

Conventional microbial detection techniques are costly, time-consuming and require specialized equipment. This research demonstrates a nuclear magnetic resonance method for rapid, sensitive and highly specific detection of *V. parahaemolyticus* in shrimp tissue, allowing rapid response to public health concerns compared to conventional detection techniques.

## Nlrx1, a mitochondrial Nlr protein

Innate immunity has evolved mechanisms to detect and respond to conserved danger-associated molecular patterns (DAMPs) and microbial-associated molecular patterns (MAMPs) [1]. Common examples of DAMPs include adenosine triphosphate (ATP), potassium efflux from the plasma membrane, but also damages to cellular organelles, such as lysosomal membrane damage or mitochondrial stress, although in the latter cases, the molecular underpinnings and the nature of the exact DAMPs remain poorly understood. As for MAMPs, the most studied include lipopolysaccharide (LPS), peptidoglycan, flagellin and bacterial/viral nucleic acids. In mammals, MAMPs and DAMPs are detected by an arsenal of germline-encoded sensors, also known as pattern recognition molecules (PRMs) [1]. Membrane-bound PRMs include Toll-like receptors (TLRs), Dectin-1, scavenger and mannose receptors, which provide extracellular pattern detection. In addition, there are several families of intracellular PRMs, which specifically detect DAMPs and MAMPs once they get access to the intracellular compartment. Intracellular PRM families include the Nod-like receptors (NLRs), Rig-I-like receptors (RLRs), Aim2-like receptors (ALRs), Protein kinase R (PKR), the cGAS/STING pathway and the oligoadenylate synthase (OAS)/RNAse L system.

**Fig. 1:**
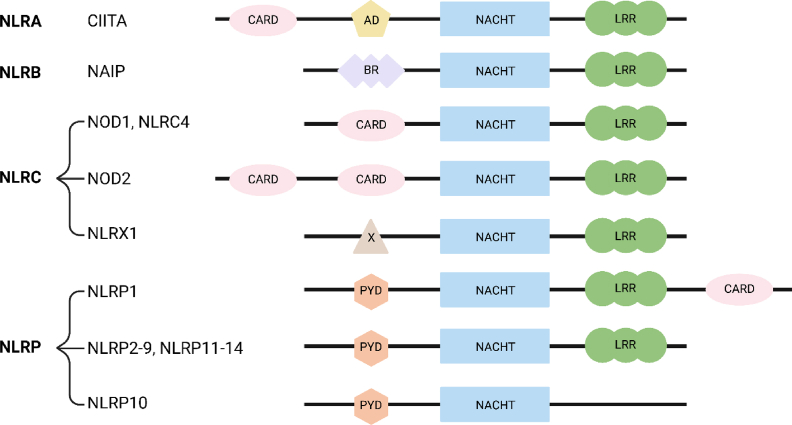
**The NLR family**. The human NLR family can be divided into four sub-families: NLRA, NLRB, NLRC and NLRP. All human NLR proteins contain a central NACHT domain and a C-terminus LRR domain, with the exception of NLRP10, which lacks the C-terminus LRR domain. The N-terminus domain of NLR is variable, and the nomenclature of each domain is abbreviated as below: Abbreviations: ATD: acidic transactivation domain; BR: Baculoviral inhibition of apoptosis protein repeat domain; CARD: caspase association and recruitment domain; FIIND: function to find domain; LRR: leucine-rich repeats; NACHT: NAIP (neuronal apoptosis inhibitor protein), C2TA (MHC class 2 transcription activator), HET-E (incompatibility locus protein from *Podospora anserina*) and TP1 (telomerase-associated protein) domain; PYD: pyrin domain; X: undefined domain.

In mammals, the NLR family is composed of approximately 20 members, the exact number varying between species as a result of gene deletion or duplication [2,3]. NLRs share a similar modular domain organization with a central NACHT domain (the acronym NACHT stems from being first identified in the proteins NAIP, CIITA, HET-E and TEP1) important for protein oligomerization and a C-terminal leucin-rich repeat (LRR) region typically involved in pattern detection [Fig. 1]. Some of the diversity in the NLR family comes from the N-terminal region, which can include caspase-activation and recruitment domain (CARD) or Pyrin domains; additionally, three NLR proteins (NLRX1, NLRC3 and NLRC5) have unique and dissimilar N-terminal regions [Fig. 1].

Early work on NLRX1 identified that the unusual N-terminal region of the protein encodes a canonical mitochondrial targeting sequence (MTS), which directs the protein to cross the translocase of outer membrane (TOM) and the translocase of the inner membrane (TIM) complexes of the mitochondria, to reach the matrix [4,5]. NLRX1 MTS was shown to be functional for targeting the protein since an NLRX1 construct lacking the MTS remained fully cytosolic [4,5]. In the matrix, the first 39 amino acids of NLRX1's N-terminal region are removed by matrix proteases, giving rise to the mature protein [5]. In this location, NLRX1 interacts with UQCRC2 [5,6], UQCRC2, a matrix-facing protein of the respiratory chain complex III, which was proposed to impact on the generation of mitochondrial reactive oxygen species (ROS). Early studies also proposed that over-expressed NLRX1 localized to the mitochondrial outer membrane, facing the cytosolic side, which was surprising and unusual for mitochondrial proteins expressing a functional MTS [7]. In this location, as discussed further below, NLRX1 was proposed to bind and inhibit the function of MAVS, a critical adaptor of the RIG-I/MDA-5 antiviral pathway. However, subsequent studies analyzing the sub-cellular localization of the endogenous form of NLRX1 failed to identify accumulation of the protein on the cytosolic side of the MOM, at least in resting conditions [5].

## Cellular function of Nlrx1

As part of the NLR family, NLRX1 characteristically regulates innate immune system function. However, its unique mitochondrial localization also allows it to control processes not traditionally associated with NLRs. Interestingly, NLRX1 has been shown to regulate various mitochondrial homeostatic processes, including metabolism [[8], [9], [10]] and cell death [[11], [12], [13], [14]] to potentially control inflammation. Moreover, studies have identified a number of proteins that interact with NLRX1, either in the mitochondrial matrix or in the cytosol (reviewed elsewhere [15]), which suggest that NLRX1 may regulate multiple cellular processes. Here, we will explore the various cellular functions associated with NLRX1 and the mechanisms by which such regulation occurs.

### Regulation of innate immunity

The innate immune system relies on a diverse repertoire of PRMs to recognize and respond to pathogens. Several studies over the past 15 years suggest that NLRX1 regulates innate antiviral responses by disrupting multiple crucial PRM signalling pathways that induce pro-inflammatory cytokines and antiviral mediators. *In vitro* studies first identified that NLRX1 interacts with MAVS, an outer mitochondrial membrane adaptor protein that associates with RIG-I and MDA5 to induce type I interferon and NF-κB responses to RNA viruses. NLRX1 was proposed to sequester MAVS by preventing RIG-I/MAVS association, thereby dampening the antiviral response [7]. NLRX1 was also shown to activate poly (rC) binding protein 2 (PCBP2) to facilitate proteasomal degradation of MAVS, further inhibiting RIG-I/MAVS signalling and subsequent type I IFN production [16]. In line with these studies, NLRX1-deficient cells infected with Sendai and Hepatitis C virus, both RNA viruses known to activate the RIG-I/MAVS pathway, displayed greater IFN-β production [7,16]. Interestingly, NLRX1-dependent regulation was also observed in DNA virus infections, not commonly known to trigger RIG-I/MAVS. To explain this, mechanistical studies suggested that NLRX1 may prevent STING/TBK1 association, much like RIG-I/MAVS, to decrease type I IFN production [17]. NLRX1 as a negative regulator of antiviral immunity was recapitulated in *Nlrx1*^*−/−*^ mice infected with Influenza virus, denoted by increased IFN-β and inflammation compared to their WT counterparts [18]. In addition to inhibiting RIG-I and STING, NLRX1 was also shown to diminish NF-κB levels by associating with tumor necrosis factor receptor associated factor 6 (TRAF6) to prevent TLR signalling, further dampening antiviral responses [18,19].

Contrastingly, there is evidence that NLRX1 may not negatively regulate the antiviral response [6,20,21]. NLRX1-deficient murine embryonic fibroblasts (MEFs) and mice challenged with poly (I:C), a viral mimetic displayed no alterations in IFN-β levels [21]. Interestingly, NLRX1/MAVS interactions could not be detected [6]. Furthermore, *Nlrx1*^*−/−*^ mice exposed to Influenza A also failed to elicit an altered antiviral response, confirming that the effect was not an artifact of using a viral mimetic [21]. In fact, overexpression of NLRX1 increased intracellular ROS and induced the NF-κB and JNK pathways suggesting that NLRX1 may even strengthen the antiviral response, at least in some conditions [4]. In line with this, polymerase basic protein 1-frame 2 (PB1–F2) encoded by Influenza A was shown to bind to NLRX1 and stimulate type I IFN synthesis [12]. These conflicting data suggest that NLRX1-associated antiviral regulation may be possibly highly dependent on viruses and host cell types. Alternatively, NLRX1 may have opposing regulatory effects on interferon regulatory transcription factors (IRFs). For instance, in hepatocytes NLRX1 supressed MAVS dependent IRF3 activation, but activated the IRF1 antiviral response by inhibiting protein kinase R (PKR) from halting translation [22].

Beyond regulating antiviral immunity, NLRX1 may also play a critical role in regulating antibacterial immunity. While NLRX1 limited Group A *Streptococcus* invasion and autophagy [30,488,027], it increased *Helicobacter pylori* burden in vivo [23] signifying multiple layers of regulation. Similarly, NLRX1-dependent regulation of ROS levels was critical for allowing the growth of *Chlamydia trachomatis* [24]. Recently, VgrG4, a type VI secretion system effector encoded by *K. pneumoniae,* was shown to decrease NF-κB activation in an NLRX1-dependent manner, thereby highlighting a novel immune evasion mechanism [25]. Collectively, the aforementioned studies suggest that NLRX1 may regulate innate immunity through multiple facets.

### Cellular metabolism

Mitochondria are highly dynamic, undergoing constant fusion and fission. Abnormal mitochondrial dynamics has long been associated with bioenergetic impairment [26]. Though NLRX1 is a PRM, it may also play a critical in maintaining mitochondrial homeostasis. In N2A cells, NLRX1 knock-in resulted in excessive mitochondrial fission and abnormal mitochondrial morphology denoted by swollen cristae [27], suggesting that NLRX1 may regulate mitochondria-associated metabolic processes. Indeed, early studies in cancer cells demonstrated that NLRX1 promoted glycolysis [13] and displayed glucose-dependent expression [11]. Even in non-cancerous hepatocytes, NLRX1 increased glycolysis while reducing oxidative phosphorylation (OXPHOS) [10]. Mechanistically, NLRX1 may decrease OXPHOS by reducing complex III activity in the mitochondrial respiratory chain (MRC) through interactions with UQCRC2, a subunit of complex III [5,6], although the functional impact of NLRX1/UQCRC2 on mitochondrial function has not yet been directly investigated. Additionally, the LRR of NLRX1 associated with Fas-activated serine–threonine kinase family protein-5 (FASTKD5) to disrupt FASTKD5-mediated processing of mitochondrial RNA, especially transcripts required for complex I and IV of the MRC [28]. Disruption of complex I, III, and IV impairs normal oxidative phosphorylation, thereby reducing OXPHOS. Thus, NLRX1 may modulate the OXPHOS through multiple pathways.

Interestingly, NLRX1 regulation of OXPHOS seems to be cell type dependent. As mentioned above, NLRX1 inhibited OXPHOS and promoted glycolysis in cancer and non-immune cells; however, NLRX1 increased OXPHOS in immune cells. In CD4+ T cells, NLRX1 deficiency decreased OXPHOS and led to Th17 and Th1 differentiation by promoting aerobic glycolysis in models of colitis induced by dextran sodium sulfate (DSS) [29].

Beyond regulating energy metabolism, NLRX1 was also proposed to control amino acid metabolism. *Nlrx1*^*−/−*^ intestinal epithelial cells and organoids displayed upregulated glutamine metabolism genes and greater glutamate dehydrogenase activity respectively [30]. Furthermore, NLRX1 maintained glutamate homeostasis in the central nervous system (CNS) by stimulating glutamate uptake and inhibiting Ca^2+^ mediated glutamate exocytosis in astrocytes [31]. By regulating amino acid metabolism in the gut and CNS, NLRX1 may dampen inflammatory responses by modulating the microbiome and neuronal death respectively [30,31]. As the field of immunometabolism continues to explore how alterations in metabolism can regulate inflammatory responses, NLRX1 will undoubtedly be at the forefront of such future studies.

### Cell death

In addition to energy metabolism, mitochondria play a crucial role in regulating cell death. ROS, a common by-product of mitochondrial metabolism, is known to regulate cell death processes [32]. In support, various studies have demonstrated that NLRX1 increases ROS levels [4,33], and this was shown to induce subsequent cell death in auditory cells [34]. Moreover, NLRX1 activated caspase-8 and stimulated ROS production, thus sensitizing the cell to TNF-induced apoptosis [13]. As such, NLRX1 displayed tumor suppressor characteristics. Indeed, NLRX1 reduced tumor progression in colitis [11] and sarcoma models [35].

Conversely, other data suggests that NLRX1 may inhibit cell death. For instance, NLRX1 protected cells from apoptosis by decreasing ROS levels in tubular epithelial cells [8]. NLRX1 also directly inhibited virally induced apoptosis in macrophages during Influenza A infection by binding to virus-encoded PB1–F2 [12]. Discrepancies in data surrounding NLRX1 cell death mediation may occur due to opposing regulation on the intrinsic and extrinsic apoptosis pathways. More specifically, NLRX1 in SV40-transformed MEFs promoted intrinsic apoptosis while supressing extrinsic apoptosis signals [11]. Recent mechanistical studies demonstrated that this extrinsic and intrinsic apoptosis regulation occurs via NLRX1 interactions with sterile alpha and Toll/interleukin-1 receptor motif-containing protein 1 (SARM1) [36]. Future studies will be required to further clarify the underlying mechanisms of cell death regulation by NLRX1.

Thus, despite being classified as a PRM, NLRX1 evidently appears to regulate a myriad of mitochondrial functions including metabolism and cell death. Current evidence suggests that cell type, microenvironment, and stimulus all dictate the regulatory impact of NLRX1 on various processes, suggesting that NLRX1 may ultimately facilitate the maintenance of cellular homeostasis. All these apparently divergent functions of NLRX1 may also suggest that the true, overarching function of this protein may be to control a unique essential cellular process, and that all the main diverse functions, including regulation of antiviral immunity, metabolism and cell death, might be secondary to the impact of NLRX1 on this main cellular process. However, until recently, the nature of this hypothetical overarching process regulated by NLRX1 had remained elusive. As detailed in the next section, the most recent work on NLRX1 has now suggested that NLRX1 regulates mitophagy, which might represent this elusive overarching cellular process long sought after.

### Mitophagy

Through continued investigation, NLRX1 has been identified as a major player in the quality control pathway of mitochondrial autophagy, known as mitophagy [[37], [38], [39], [40], [41], [42]]. Mitophagy involves the sequential labeling and engulfment of mitochondria in double membrane lipid phagophores, that ultimately get degraded upon fusion with lysosomes [43]. The subset of mitochondria designated for removal are labelled with an “eat-me” signal such as ubiquitination, or accumulation of an LC3-interacting region (LIR) motif containing mitophagy receptor. This selective degradation of damaged or ineffective subregions ensures homeostasis of the network as a whole, and a number of recent reviews thoroughly cover the molecular regulators and physiological implications of mitophagy [43,44]. In 2019, Zhang et al. demonstrated that mitophagy is induced by *Listeria* infection [37]. This process occurred downstream of the *Listeria* toxin Listeriolysin O (LLO), in a manner that limited mitochondrial ROS to promote bacterial infection. The authors placed NLRX1 as the central mediator of this form of mitophagy and characterized a LIR motif within the central NACHT domain of NLRX1 that was essential for the binding and recruitment of LC3 to mitochondria. This finding positions NLRX1 in a similar category of LIR-containing mitophagy receptor as BNIP3, NIX and FUNDC1. Similar to other NLRs, NLRX1 formed high molecular weight oligomers in response to mitochondrial stress, which may be necessary for mediating mitophagy progression. An explanation for how NLRX1, a protein found in the mitochondrial matrix, was able to facilitate local LC3 binding and phagophore expansion around the outside of the mitochondria remained to be discovered.

**Fig. 2:**
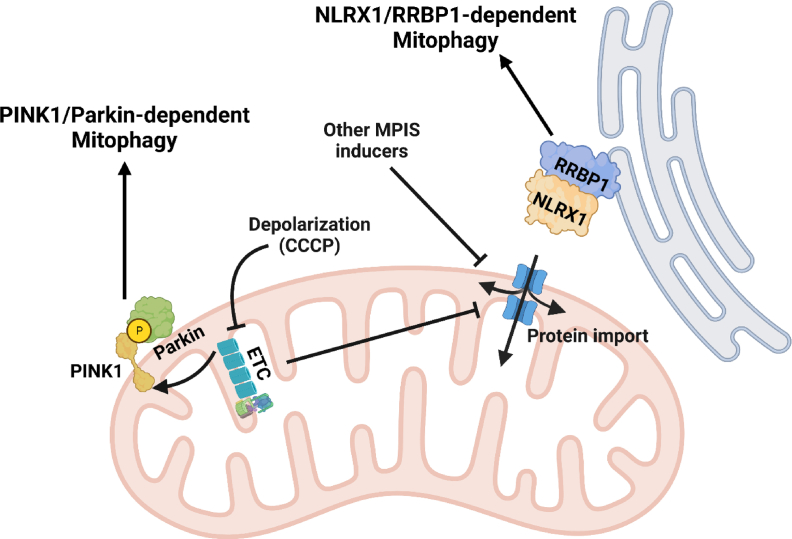
**NLRX1-RRBP1 directed mitophagy.** When mitochondrial membrane potential is disrupted by ionophores such as CCCP or other depolarizing agents, PINK1 is stabilized on the outer membrane. PINK1 then recruits Parkin, which subsequently labelled the dysfunctional mitochondria for mitophagy. Mitochondrial depolarization also results in mitochondrial protein import stress (MPIS), which leads to cytosolic retention of NLRX1. Cytosolic NLRX1 then interacts with endoplasmic reticulum (ER) protein RRBP1 and together they induce mitophagy by facilitating the recrement and lipidation of LC3 around the damaged mitochondria. Abbreviations: CCCP: carbonyl cyanide m-chlorophenyl hydrazone; ETC: electron transport chain; NLRX1: NLR family member X1; MPIS: mitochondrial protein import stress; PINK1: PTEN-induced kinase 1; RRBP1: ribosome-binding protein 1. Created on biorender.com.

To understand the mechanism of NLRX1-mediated mitophagy, we followed this open question and first assessed the subcellular localization of NLRX1 during diverse mitochondrial stress. We were surprised to see that NLRX1 was retained and stabilized in the cytosol upon mitochondrial depolarization, a common approach to induce widespread mitophagy [40]. Interestingly, we also detected high molecular weight oligomers of NLRX1 during mitophagy, further supporting this characteristic of NLRX1 activation. Aside from depolarization, our study implicated NLRX1 in mitophagy downstream of various other mitochondrial stressors with seemingly disparate mechanisms of action. This led to the major conceptual advancement that mitochondrial protein import stress (MPIS) is the unifying signal for mitophagy (Fig. 2). This model also answered how a mitochondrial matrix protein could be interacting with LC3. Since NLRX1 is synthesized in the cytosol, upon import disruption, NLRX1 was not reaching the mitochondrial matrix, and instead was serving as a local signal to reflect defective import. Disrupting the efficient and tightly regulated import of nuclear-encoded mitochondrial precursor proteins triggers mitophagy initiation through the cytosolic retention of NLRX1 and subsequent LC3 recruitment, lipidation and phagophore expansion around import-deficient mitochondria.

The presence of NLRX1 outside of the mitochondria raised the question of how the retained NLRX1 serves as a local signal at the site of disrupted protein import, rather than diffusing nonspecifically into the cytosol. By examining the interacting partners of NLRX1, the endoplasmic reticulum transmembrane protein RRBP1 was identified as the missing link required for LC3 lipidation [40]. We showed that the retained, cytosolic NLRX1 interacts with RRBP1, and that RRBP1 was necessary for MPIS-driven mitophagy [Fig. 2]. Importantly, this NLRX1-RRBP1 driven LC3 lipidation was independent of the commonly studied PINK1-Parkin pathway of mitochondrial labelling with ubiquitination, although final degradation of mitochondria was more efficient when PINK1-Parkin were active, suggesting two parallel processes that cooperate in targeting and removing damaged mitochondria.

The physiological relevance of NLRX1-driven mitophagy was further demonstrated in vivo using NLRX1 full body knockout (KO) mice. Over recent years, the field has begun to induce mitophagy in various tissues by pushing mice to exhaustion through forced exercise [45,46]. In these settings, mitophagy deficient animals succumb to exhaustion earlier than their littermate control, likely due to the suboptimal metabolic capacity and oxygen utilization of the mitochondrial network. Upon forced exercise, NLRX1-deficient animals exhibited this reduced endurance capacity, in combination with decreased mitochondrial LC3 lipidation in lysates taken from the gastrocnemius muscle [40].

Aside from these aforementioned papers, recent studies bring additional support to the importance of NLRX1 in mitophagy following diverse physiological conditions, and in a range of cell types. Of note, NLRX1-driven mitophagy was demonstrated in response to morphine stimulation in microglia, to hypoxic-ischemic brain damage and ischemia-reperfusion injury in intestinal tissue [38,41,47]. In addition, deficiencies in phospholipase A-2-activating protein (PLAA) disrupts the proteasome-based removal of mitochondrial proteins like MCL1, an event which was placed upstream of high molecular weight oligomers of NLRX1 and mitophagy induction [42]. The close agreement in molecular characterization from multiple groups helps position NLRX1 as one of the key drivers of mitophagy and mitochondrial homeostasis across different tissues and conditions. In future studies, it will be important to determine if mitophagy is indeed the over-arching NLRX1-dependent cellular process that in turns dictates the regulation of antiviral responses, cell death and metabolism. This would help resolve the conundrum of how this protein could display so many apparently distinct and divergent functions in the cell.

## Nlrx1 in diseases

Dysfunction and dysregulation of the mitochondrial system have been linked to the pathogenesis of multiple human diseases, such as CNS disorders, cardiovascular diseases, and cancer [48]. In addition, the importance of mitochondria has also been recognised in the regulation of innate and adaptive immunity [49]. As the only known NLR that locates to the mitochondria [5], NLRX1 became a focus of research due to its unique biology. Next, we will discuss recent studies that investigated the role of NLRX1 in the pathology of mitochondria-related diseases and the potential of NLRX1 as a target for therapeutics.

### Inflammatory disorders

Members of NLR family are important regulators of the inflammatory response and were involved in various inflammatory disorders such as Multiple sclerosis (MS) [50] and inflammatory bowel disease (IBD) [2]. MS is a chronic inflammatory disorder in the CNS that is characterized by neuronal loss resulting from demyelination and autoimmune attack [51]. Mitochondrial dysfunction has been implicated in the development of MS [52] and rare mutations in the *NLRX1* gene have been identified in MS patients with family history [53]. To study the involvement of NLRX1 in MS, multiple studies utilized a murine model of experimental autoimmune encephalomyelitis (EAE) that mimicked the onset of MS [53,54]. Consistent to previous *in vitro* studies, NLRX1 was found to be immunosuppressive and anti-inflammatory, and overall played a protective role during EAE [53,54]. Compared to WT mice, *Nlrx1*^−/−^ mice expressed more pro-inflammatory cytokines, accumulated more encephalitogenic T cells and contained hyperactive microglial cells in the CNS tissues [53,54]. These mice also suffered aggravated neuronal demyelination and paralysis in limbs, which let to significantly worsened clinical scores and outcomes [53,54]. To investigate the potential of NLRX1 as a therapeutic target for MS, a recombinant form of NLRX1 was created by fusing the LRR region of NLRX1 with a blood brain barrier (BBB)-penetrable peptide dNP2 [55]. Following the injection of the NLRX1-LRR fusion protein, EAE mice showed overall reduced disease severity, with less T cell infiltration and decreased levels of inflammatory cytokines in the spinal cord tissues [55].

IBD is a group of chronic inflammatory disorders that affects the gastrointestinal tract, which includes Crohn's disease (CD) and ulcerative colitis (UC) as the principal types [2]. While the etiology of IBD remained unclear to this day, recent evidence has indicated mitochondrial dysfunction as one of the potential contributing factors of IBD [56]. DSS-induced colitis is a well-accepted model of IBD in mice, and when *Nlrx1*^*−/−*^ mice were challenged with acute DSS treatment, increased T cell population and elevated levels of pro-inflammatory cytokines were observed in the colon, and these mice generally suffered worsened disease manifestation compared to the control group [29]. Moreover, mice with T cell-specific *Nlrx1* deletion (*Nlrx1*^ΔT^) also experienced aggravated disease severity and adoptive transfer of *Nlrx1*^*−/−*^ CD4+ T cells in WT mice led to exacerbated local and systemic inflammation [29]. At the cellular level, NLRX1 deletion was shown to alter T cell metabolism and induce T cell proliferation and differentiation [29]. Once the link between NLRX1 and the development of IBD was established, several studies explored the feasibility of targeting NLRX1 in the treatment of IBD by testing a small molecule NLRX1 activator named NX-13 [57,58]. Repeated dosage of NX-13 in rats were found to be well-tolerated and gastrointestinal localization of the drug was also confirmed [58]. Oral treatment with NX-13 in DSS-challenged mice resulted in decreased infiltration of immune cells in colon, as well as reduced levels of inflammation markers and overall disease severity [57]. Similar results were observed in additional murine IBD models such as the CD45RB^hi^ adoptive transfer model and *Mdr1a*^−/−^ model [57]. In primary PBMCs collected from UC patients, artificial activation of NLRX1 again showed promising therapeutic benefits as NX-13 treatment blunted NF-κB activity, cytokine production and T cell differentiation [57].

Collectively, these studies proposed a protective role for NLRX1 in various inflammatory disorders and provide insight in the development of novel therapeutics that target NLRX1.

### Ischemia-Reperfusion Injury

Ischemia-Reperfusion Injury (IRI) occurs when blood flow returns to tissues that experienced a stage of ischemia and hypoxia, and it is most commonly associated with conditions such as myocardial infraction (MI) and stroke [59]. The tissue damage from IRI is strongly linked to mitochondrial dysfunction and accumulation of ROS during the hypoxia period [60]. The expression of NLRX1 was down-regulated in human cardiac tissues following acute myocardial infraction (AMI), as well as rat heart-derived H9c2 cells after hypoxia challenge [61]. Similar to MS and IBD, the role played by NLRX1 in IRI was found to be protective. When the level of NLRX1 was restored by over-expression, the level of hypoxia-induced pro-inflammatory cytokine production and apoptosis were significantly reduced in H9c2 cells, via a NLRX1-mediated block on the activation of MAVS-directed NLRP3 inflammasome [61]. To further elucidate the role of NLRX1 during IRI, a recent report utilized a murine model of MI and discovered that *Nlrx1*^−/−^ mice also experienced signs of worsened cardiac injury following reperfusion [62]. During initial period of reperfusion, the activation of the pro-survival Akt signaling was blunted in the hearts of *Nlrx1*^−/−^ mice [62]. After continued reperfusion, lactose accumulation, glucose oxidation, and oxygen consumption in *Nlrx1*^−/−^ heart were all found to be elevated compared to wild type control, suggesting a regulatory role of NLRX1 in the energy metabolism of the heart [62]. In addition to cardiac IRI, NLRX1 was also implicated in the progression of IRI of the brain according to results from cellular and murine models of cerebral ischemia. In cells collected from the infarct regions of the brain, upregulation of DJ-1 mediated the dissociation of NLRX1 from TRAF6 [63]. NLRX1 then inhibited the production of pro-inflammatory cytokines such as IL-1β, IL-6 and TNF-α via the negative regulation that it exhibited on NF-κB signaling [18,19,63].

Overall, results from these studies illustrated the protective role of NLRX1 in IRI and demonstrated the potential of targeting regulators of NLRX1 such as DJ-1 in the development of novel therapeutics of IRI in various tissues.

### Cancer

Members of the NLR family have been increasingly recognized as important regulators of cancer development, particularly due to their critical roles in the mediation of innate immunity, inflammatory response and programmed cell death [64]. Typically, NLRX1 displayed the property of a tumor suppressor and the expression level of NLRX1 was blunted in multiple types of cancers [35,[65], [66], [67]]. In DSS/AOM and *APC*^*min/+*^ murine models of colorectal cancer (CRC), NLRX1 was shown to supress the development of CRC, as deletion of NLRX1 resulted in increased susceptibility, mortality and tumor burden [14,66]. Specifically, oncogenic pathways that drive cell proliferation, such as NF-κB, MAPK and IL-6/STAT3 pathways showed elevated activity in *Nlrx1*^*−/−*^ mice [66]. Conversely, the introduction of an antibody that targets IL-6 receptor led to blunted activation of STAT3 and decreased tumor burden, which overall promoted survival in the *Nlrx1*^−/−^*Apc*^*min*/+^ mice [66]. In addition, mice with intestinal epithelial cell (IEC)-specific deletion of *Nlrx1* (*Nlrx1*^ΔIEC^) also showed up-regulation of genes that are involved in tissue repair and healing, such as *Tnf*, *Egf*, and *Tgfb1*, as well as cell proliferation marker Ki-67, following challenge of DSS [14]. Furthermore, following stimulation with TNF, intestinal organoid developed from *Nlrx1*^ΔIEC^ mice displayed elevated expression levels of transcription factor *Myb* and transit-amplifying (TA) zone stem cell marker *Olfm4*, which have been linked to the development of cancer [68]. Besides CRC, NLRX1 was also shown to inhibit the development of other types of cancers, such as histolytic sarcoma [35] and hepatocellular carcinoma [65].

In summary, these results highlighted the importance of NLRX1 as a tumor suppressor gene and introduced innovative cancer therapy strategies that may target NLRX1-regulated oncogenic pathways.

## Concluding remarks

NLRX1 is a unique protein in that it is one of the only PRM proteins, if not the only PRM, which targets the mitochondrial matrix. Since PRM proteins typically detect either microbial molecules or specific danger signals, it is not surprising that NLRX1 may have evolved to monitor and control specific processes associated with the mitochondria, by detecting a critical mitochondria-specific danger signal. For many years, the nature of this signal (or of these signals if NLRX1 was able to detect multiple cues) had remained hotly debated and elusive. A key difficulty in assessing the true signal detected by NLRX1 has always been to define if the observed effects were direct or were downstream of another, more general, cue. For instance, does NLRX1 directly control apoptotic cell death, or is the cell death an indirect consequence of something else that NLRX1 controls? Or, similarly, does NLRX1 directly control mitochondrial metabolism from within the matrix sub-compartment, or does it do so indirectly as a consequence of more fundamental effects that modulate mitochondrial fitness or mitochondrial physiology (for instance the fusion/fission machineries)? Does NLRX1 regulate antiviral immunity through a direct binding with MAVS at the OMM or is the antiviral MAVS-dependent function indirectly modulated by more global changes in mitochondrial function? In support of the latter, multiple lines of evidence argue that MAVS-dependent signaling is indeed tightly controlled by mitochondrial physiology, for instance the fusion/fission machinery [69]. The recent emergence of the notion that NLRX1 controls mitophagy in multiple experimental settings suggests that this cellular process might represent the overarching common mechanism through which NLRX1 may display so many apparently divergent cellular functions. Indeed, defective mitophagy that would occur in the absence of proper control by NLRX1 would result in the accumulation of dysfunctional and older mitochondria, which in turn could impact on multiple downstream pathways. Furthermore, our recent work has suggested that the molecular cue (or “danger”) that NLRX1 detects to control mitophagy processes, was the defective import of proteins into the mitochondria, or MPIS (see the “*Mitophagy*” section above). The characterization of the molecular underpinnings of MPIS and mitophagy regulation by NLRX1 will provide opportunities to rationally design novel therapeutics aiming at improving mitochondrial function, which may be of critical importance for numerous pathologies, including neurodegenerative diseases, cancer, cardiac and inflammatory diseases.

## Declaration of competing interest

The authors declare no conflict of interest.
